# Prediction of potential drug targets and key inhibitors (ZINC67974679, ZINC67982856, and ZINC05668040) against *Rickettsia felis* using integrated computational approaches

**DOI:** 10.3389/fvets.2024.1507496

**Published:** 2025-01-16

**Authors:** Sudais Rahman, Hsien Liu, Mohibuallah Shah, Mashal M. Almutairi, Iram Liaqat, Tetsuya Tanaka, Chien-Chin Chen, Abdulaziz Alouffi, Abid Ali

**Affiliations:** ^1^Department of Zoology, Abdul Wali Khan University, Mardan, Khyber Pakhtunkhwa, Pakistan; ^2^Division of General Surgery, Department of Surgery, Ditmanson Medical Foundation Chia-Yi Christian Hospital, Chiayi, Taiwan; ^3^Department of Biochemistry, Bahauddin Zakariya University, Multan, Pakistan; ^4^Department of Pharmacology and Toxicology, College of Pharmacy, King Saud University, Riyadh, Saudi Arabia; ^5^Microbiology Lab, Department of Zoology, Government College University Lahore, Lahore, Pakistan; ^6^Laboratory of Animal Microbiology, Faculty of Agriculture, Graduate School of Agricultural Science, Tohoku University, Sendai, Japan; ^7^Department of Pathology, Ditmanson Medical Foundation Chia-Yi Christian Hospital, Chiayi, Taiwan; ^8^Department of Cosmetic Science, Chia Nan University of Pharmacy and Science, Tainan, Taiwan; ^9^Doctoral Program in Translational Medicine, National Chung Hsing University, Taichung, Taiwan; ^10^Department of Biotechnology and Bioindustry Sciences, College of Bioscience and Biotechnology, National Cheng Kung University, Tainan, Taiwan; ^11^King Abdulaziz City for Science and Technology, Riyadh, Saudi Arabia

**Keywords:** *Rickettsia felis*, novel drug targets, succinate dehydrogenase, *in silico* screening, pharmacokinetics

## Abstract

*Rickettsia felis*, responsible for flea-borne spotted fever, is a rising zoonotic pathogen posing an increasing global threat due to its expanding geographical distribution. The rise in antibiotic-resistant strains of this pathogen underscores the urgent need for new therapeutic interventions. This study employed a comprehensive subtractive proteomics analysis of the *R. felis* proteome, aiming to identify essential, non-host homologous, and pathogen-specific proteins, which were subsequently evaluated as potential new drug targets. These findings offer valuable insights into the development of therapeutic strategies against rickettsiosis. The analysis revealed 343 proteins that are non-homologous to the host, including 108 essential proteins, 25 unique metabolic pathways, and 11 distinct proteins. Out of these, 10 proteins were druggable in which two associated with virulence, and one related to resistance (succinate dehydrogenase). Through a rigorous screening process and extensive literature review, succinate dehydrogenase emerged as a promising drug target. Protein interaction partners for succinate dehydrogenase were identified using the STRING database. To further assess the functionality of succinate dehydrogenase, structure-based studies were conducted. Approximately 18,000 ZINC compounds were screened, leading to the finding of six potential inhibitors: ZINC67847806, ZINC67982856, ZINC67974679, ZINC67895371, ZINC05668040, and ZINC05670149. Absorption, distribution, metabolism, excretion, and toxicity (ADMET) profiling confirmed that most compounds met the preferred pharmacokinetic properties, except for ZINC67895371 and ZINC67847806, which exhibited positive ames test results, and ZINC05670149, ZINC67895371, and ZINC67847806, showed hepatotoxicity. All compounds were found to be non-sensitizing to the skin. Based on these findings, further experimental validation of ZINC67974679, ZINC67982856, and ZINC05668040 is recommended.

## 1 Introduction

*Rickettsia felis* is a gram-negative, obligate intracellular bacterium that causes flea-borne spotted fever (1). *R. felis* is a rod-shaped bacterium, usually around 0.3 to 0.5 micrometers wide and 0.8–1.5 micrometers long (2). It possesses a rickettsial cell wall structure, with a peptidoglycan layer and an outer membrane that is essential for its ability to survive and cause disease in the host (3). *R. felis* was first discovered in 1994 in cat fleas, known as *Ctenocephalides felis*, and has since been acknowledged as a new zoonotic agent found worldwide (4). It has been found in different arthropod vectors, such as fleas (5, 6), ticks (7, 8), and mites (9), as well as in a variety of mammals hosts (10), indicating a wide ecological range. The global distribution of *R. felis* highlights its significance as a public health threat, particularly in underdeveloped regions where diagnostic facilities and awareness are limited (11–13). Its emergence as a zoonotic agent necessitates a deeper understanding of its epidemiology to mitigate its impact on global health. Cases of human infections have been reported globally (14), with occurrences documented in America (15–18), Europe (19, 20), Africa (21–25), and Asia (26–28). The pathogen's history of recognition is relatively recent compared to other rickettsial species (29). Its ability to infect diverse hosts and vectors, coupled with the increasing movement of pets and livestock, has facilitated its spread across different geographical regions (30–32). *R. felis* has been shown to cause pathology in various hosts, including humans, where it induces flea-borne spotted fever (33–36), as well as in animals such as cats (*Felis catus*) (37, 38), opossums (*Didelphis spp*.) (39–41), and rodents (42, 43), although the pathogenesis may differ between species due to variations in host susceptibility and immune responses. The epidemiology of *R. felis* is intricate, with numerous transmission cycles between arthropod vectors and vertebrate hosts, including humans (1, 44–49). In addition to the complexity of the life cycle, there is also the persistence and difficulty in controlling its spread. The increasing geographical spread and involvement of multiple hosts and vectors suggest that *R. felis* may pose a higher risk. Furthermore, its potential for outbreaks in densely populated areas underscores the urgency of identifying effective therapeutic targets and preventive measures (50). *R. felis* penetrates the endothelial cells lining the blood vessels, leading to a systemic infection characterized by fever, rashes, headache, and myalgia (51). Serious instances can result in complications like vasculitis and dysfunction in multiple organs (52).

The intracellular lifestyle of *R. felis* enables it to evade the host immune system, persist within host cells, and develop antibiotic resistance, rendering traditional therapies less effective and highlighting the need for novel therapeutic targets and specific inhibitors (53). *R. felis* typically shows resistance or reduced sensitivity to several classes of antibiotics, including beta-lactams (such as penicillin and cephalosporin), aminoglycosides (such as gentamicin and streptomycin), sulfonamides (such as sulfamethoxazole), fluoroquinolones, and macrolides (such as erythromycin) (54). Nonetheless, the process of evaluating numerous macromolecules followed by subsequent *in vivo* experimentation is both time-intensive and resource-draining in drug discovery (55). Subtractive proteomics, a comparative proteomics technique, allows for pinpointing essential, non-host homologous proteins crucial for the pathogen's survival (56).

Existing drugs used for treating infections can vary in their side effects in humans, and their misuse has accelerated the evolution of drug resistance in pathogens (57, 58). Subtractive proteomics is commonly used to evaluate the precision and relevance of potential therapeutic targets. It has been extensively applied in research to uncover and identify unique therapeutic targets specific to various pathogenic strains (59–62). This method ensures that identified targets are pathogen-specific, reducing the likelihood of off-target effects on the host (63). In this study, we aimed to identify potential drug targets and their inhibitors in *R. felis* using an integrated approach combining subtractive proteomics, molecular docking, virtual screening, and absorption, distribution, metabolism, and excretion (ADMET) profiling. This comprehensive strategy is designed to enhance our understanding of potential drug targets, offering promising avenues for developing effective treatments against this persistent and evolving pathogen.

## 2 Methodology

**Figure 1:**
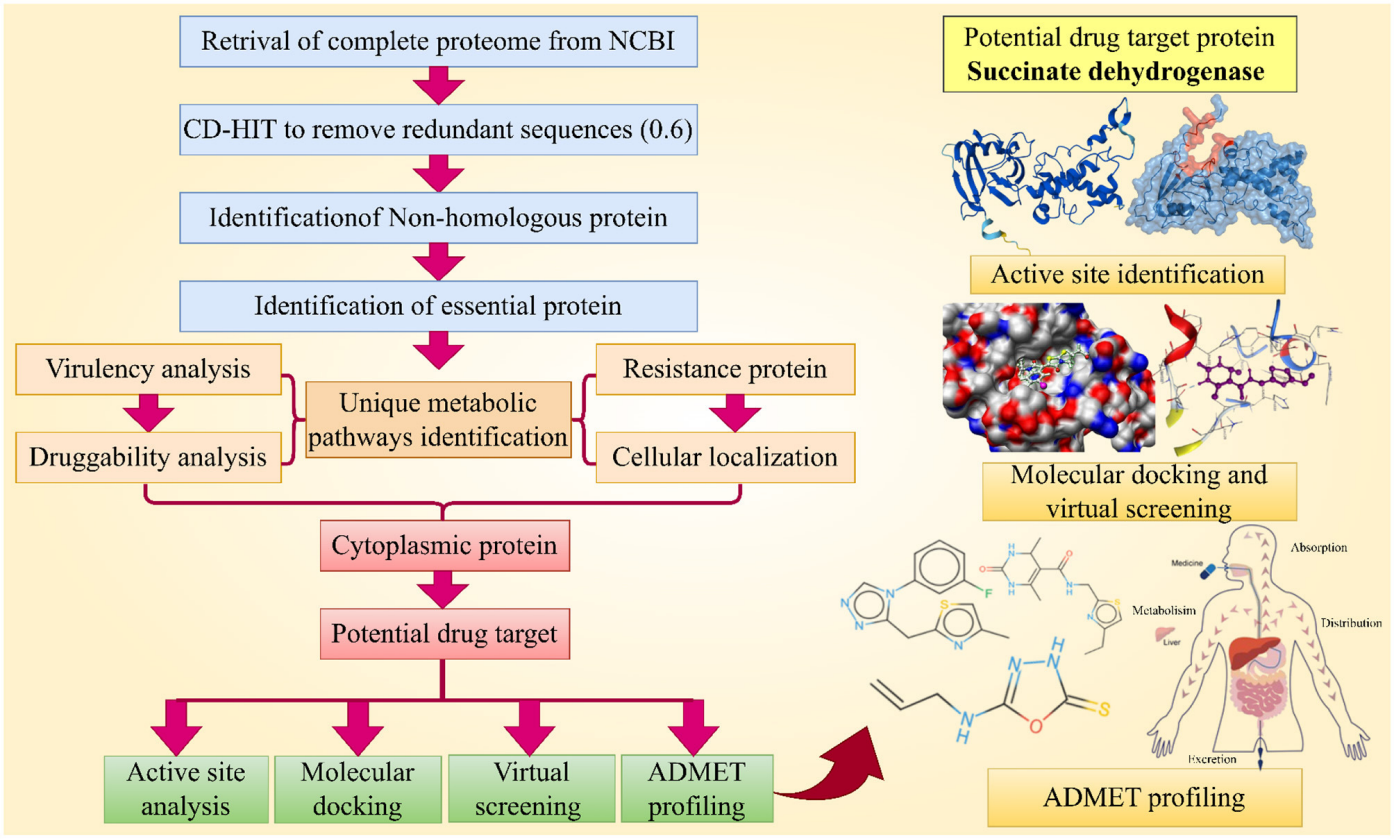
This chart illustrates the complete methodology followed for the identification of novel drug target and their inhibitors against *R. felis*. (Created with https://smart.servier.com/ and Draw.io https://app.diagrams.net/).

A subtractive proteomics approach was utilized in this study to analyze the entire proteome of *Rickettsia felis*, aiming to identify new potential drug targets, then followed by molecular docking, virtual screening and ADMET profiling which shown in Figure 1.

### 2.1 Retrieval of pathogen proteome

The entire proteome of *R. felis* was obtained from the NCBI (https://www.ncbi.nlm.nih.gov/) database in the FASTA format (Genbank ID: GCA_000804505.1, Assembly no: ASM80450v1), and was processed for the CD-HIT analysis.

### 2.2 Removal of paralogous sequences

Paralogous proteins were identified using the CD-HIT tool (64). All sequences were processed with CD-HIT, maintaining a sequence similarity cutoff of 60% to eliminate redundant proteins. Paralogue proteins were eliminated from subsequent analysis, and non-paralogue proteins comprising over 100 amino acids were chosen. Proteins with <100 amino acids were deemed non-essential and therefore excluded from the analysis.

### 2.3 Detection of host-specific protein homologs

A BLASTp search with a bit score >100 and an E-value <10^−4^ was used to identify human non-homologous proteins (65). Human homologous proteins were excluded to avoid potential drug cross-reactivity with host proteins. The non-homologous proteins with no significant similarity to human proteins were then selected for further investigation.

### 2.4 Identification of essential proteins

Essential proteins are crucial for an organism's survival, growth, and adaptability (66). Essential proteins were identified through a BLASTp search against the Database of Essential Genes (DEG) (http://tubic.tju.edu.cn/deg/), applying an E-value threshold of 10^−10^ and a bit score >100 (67). Non-essential proteins were excluded, focusing solely on proteins critical for pathogen survival.

### 2.5 Identification of unique metabolic pathways

To precisely annotate the functions of non-paralogous vital proteins, the KEGG Automatic Annotation Server (KAAS) (https://www.genome.jp/kegg/kaas/) was employed. A comparative evaluation of metabolic pathways between pathogen and host was performed using the KEGG (https://www.genome.jp/kegg/) database. KEGG used organism codes “rfe” for *R. felis* and “hsa” for *H. sapiens* to retrieve the IDs of the metabolic pathways (68). Proteins involved in pathogen-specific pathways were chosen for further identification of potential drug targets, ensuring that these targets were exclusive to the pathogen and absent in the host.

### 2.6 Druggability analysis

All essential non-homologous proteins were subsequently assessed using BLASTp to compare them with FDA-approved drug targets. The default E-value parameter of 10^−5^ was utilized in BLASTp to search the Drug Bank for potential novel drug targets.

### 2.7 Prediction of virulence protein

To assess the virulence of proteins, the VFDB (Virulence Factor Database) (http://www.mgc.ac.cn/VFs/) was utilized (69). Selected *R. felis* proteins underwent a BLASTp search against the VFDB, applying an E-value threshold of 0.0001 and a bit score above 100.

### 2.8 Identification of resistance proteins

The Antibiotic Resistance Gene Annotation Tool (ARG-ANNOT V6) tool (https://ifr48.timone.univ-mrs.fr/blast/arg-annot_v6.html) was employed to explore novel resistance proteins by analyzing the entire pathogen proteome. The FASTA sequences of the selected proteins were analyzed using a BLASTp search against the ARG-ANNOT V6 database, with a threshold of 10^−5^ (70).

### 2.9 Protein localization and interaction profiling

Proper subcellular localization is vital for protein function and interactions, influencing their role as drug or vaccine targets (71). All selected proteins were analyzed using the CELLO v.2.5 (http://cello.life.nctu.edu.tw/) online tool to determine their intracellular positioning. The position-specific iterated BLAST (PSI-BLAST) feature within this tool categorized the proteins into different subcellular compartments, including the cytoplasm, membrane, periplasmic membrane, and extracellular space (72). Additionally, the STRING (https://string-db.org/) database was utilized to identify protein-protein interactions of the identified target protein. STRING is a pre-computed database used to identify PPIs based on various data sources. To ensure the reliability of the interactions, only those with a high confidence score (≥0.7) were considered (73).

### 2.10 Protein structure modeling and validation

The 3D structure of the succinate dehydrogenase was obtained from the AlphaFold (https://alphafold.ebi.ac.uk/) database. The predicted structure included confidence scores (pLDDT) provided by AlphaFold2, with scores above 70 indicating high reliability. The quality and reliability of the predicted structure were then verified by using the Ramachandran plot and ERRAT (https://saves.mbi.ucla.edu/), ensuring the accuracy and quality of the predicted 3D model.

### 2.11 Active site analysis and ligand identification

Active site localization was accomplished using Molecular Operating Environment (MOE v. 2015) software. Forecasting active site residues was determined by pinpointing conserved sites across protein families, utilizing robust sequence-based scoring functions, and analyzing the features of the well-defined 3D structure, including the structural geometry of amino acid residues and their electrostatic and chemical properties. For ligand prediction, the computational tool ProBiS (Protein Binding Site) (http://probis.cmm.ki.si/) was employed (74).

### 2.12 Docking simulation and computational screening

The 3D structure of the protein was essential for docking studies; therefore, succinate dehydrogenase was modeled in three dimensions, and the ligand was predicted to act as an inhibitor. Docking preparation was performed for the protein-ligand complex by removing ligands and heteroatoms, including water molecules. Protein preparation was carried out using AutoDock v4.2 (75), which included adding hydrogens, merging non-polar hydrogen atoms, and assigning Kollman charges. The receptor was then saved in a local shell. Molecular docking was conducted using AutoDock with the Lamarckian Genetic Algorithm (LGA), performing 250 runs with a maximum of 27,000 generations and 2,500,000 evaluations. Redocking was performed to evaluate the program's ability to reproduce the crystal conformation of the bound ligand. The grid points on the X, Y, and Z axis were set to 64, 70, and 62, respectively. A ZINC library of 18,000 molecules was retrieved in SDF format and converted to a 3D PDB file using Open Babel. The ligand library's energy was minimized using the MMFF94 force field and the steepest descent method for 1,500 steps. Gastieger charges were added, rotatable bonds were adjusted in AutoDock, and the ligand library was saved in PDBQT format. The PDBQT library was divided into files using Vina split, with the redocking settings and grid box configuration applied for virtual screening.

### 2.13 ADMET profiling

Pharmacokinetic parameters, including absorption, distribution, metabolism, and excretion (ADME) (http://www.swissadme.ch/) were evaluated for the shortlisted drug-like compounds using the SwissADME tool (76). The pkCSM tool (https://biosig.lab.uq.edu.au/pkcsm/) was then employed to analyze the compounds' toxicity profiles, including immunotoxicity, mutagenicity, teratogenicity, neurotoxicity, increased penetration, and carcinogenicity (77). Additionally, the potential toxicity of the novel compounds was passed by evaluating the maximum tolerated dose (human), minnow toxicity, *Tetrahymena pyriformis* toxicity, acute oral toxicity (LD50) in rats, hepatotoxicity, and skin sensitization.

## 3 Result and discussion

### 3.1 Subtractive proteomics analysis

#### 3.1.1 Proteome retrieval, filtration and non-host homology analysis

The *R. felis* proteome, comprising 1,393 sequences, was subjected to CD-HIT analysis, resulting in the exclusion of 333 paralogous sequences. To prevent cross-reactivity with human proteins, the remaining 1,060 non-paralogous sequences underwent a BLASTp analysis against the *Homo sapiens* proteome (78). This analysis identified 717 sequences with human homology, which were excluded, leaving 343 non-homologous proteins for further analysis.

#### 3.1.2 Evaluation of essential proteins, unique metabolic pathways, and druggability analysis

Using DEG database, we identified 108 essential proteins in *R. felis* which are pivotal to the pathogen's life cycle and represent potential targets for antibacterial drug development. Additionally, using the KEGG database, we mapped 25 distinct metabolic pathways linked to these essential proteins. Identifying these pathways is critical as it provides insights into the metabolic dependencies, which could be exploited for therapeutic interventions. Supplementary Table S1 lists these specific pathways with their identifiers, while Supplementary Table S2 presents 11 unique proteins along with their corresponding metabolic pathways. To further assess their therapeutic potential, we performed a BLASTp search of these distinct vital proteins against the Drugbank database which revealed 10 proteins with substantial similarity to FDA-approved drug targets (Table 1). These proteins, which closely aligned with known FDA-approved drug targets, were selected for further investigation.

**Table 1:** Shortlisted druggable proteins in *R. felis*.

**Serial no**	**Protein ID**	**DrugBank target**	**DrugBank ID**	**Cellular localization**
1	WP_011270515.1	Drugbank_target | P24752 Acetyl-CoA acetyltransferase	DB00795	Cytoplasmic
2	WP_011271493.1	Drugbank_target | P0A827 Serine hydroxymethyltransferase	DB11596	Cytoplasmic
3	WP_011270920.1	Drugbank_target | P00338 L-lactate dehydrogenase A chain	DB02701; DB09118	Cytoplasmic
4	WP_011270490.1	Drugbank_target | P21912 Succinate dehydrogenase	DB00139	Cytoplasmic
5	WP_011270625.1	Drugbank_target | P04424 Argininosuccinate lyase	DB00125	Cytoplasmic
6	WP_011270995.1	Drugbank_target | P22102 Trifunctional purine biosynthetic protein adenosine-3	DB00642	Cytoplasmic
7	WP_039594975.1	Drugbank_target | P53597 Succinyl-CoA ligase	DB00139	Cytoplasmic
8	WP_039595039.1	Drugbank_target | P13995 Bifunctional methylenetetrahydrofolate dehydrogenase/cyclohydrolase	DB00116	Cytoplasmic
9	WP_039595177.1	Drugbank_target | P10902 L-aspartate oxidase	DB03147	Cytoplasmic
10	WP_011271325.1	Drugbank_target | Q02768 Cytochrome b	DB01117	Inner membrane

#### 3.1.3 Prediction of virulent and resistant proteins

Using VFDB resource, we identified two proteins from a set of 10 being associated with the virulence of *R. felis*. These proteins are of particular interest as potential drug targets, especially in the context of combating drug-resistant pathogens, which present significant challenges in the treatment and may necessitate higher doses with increased risk of adverse effects (79). Targeting virulence factors is thus a promising strategy in drug development (80). Additionally, an analysis with the ARG-ANNOT V6 tool revealed that one of the virulent proteins succinate dehydrogenase, is involved in resistance mechanisms, such as drug degradation and efflux. Despite its association with resistance, succinate dehydrogenase remains a viable drug target due to its critical role in the pathogen's biology (81).

#### 3.1.4 Subcellular localization and protein-protein interaction

**Figure 2:**
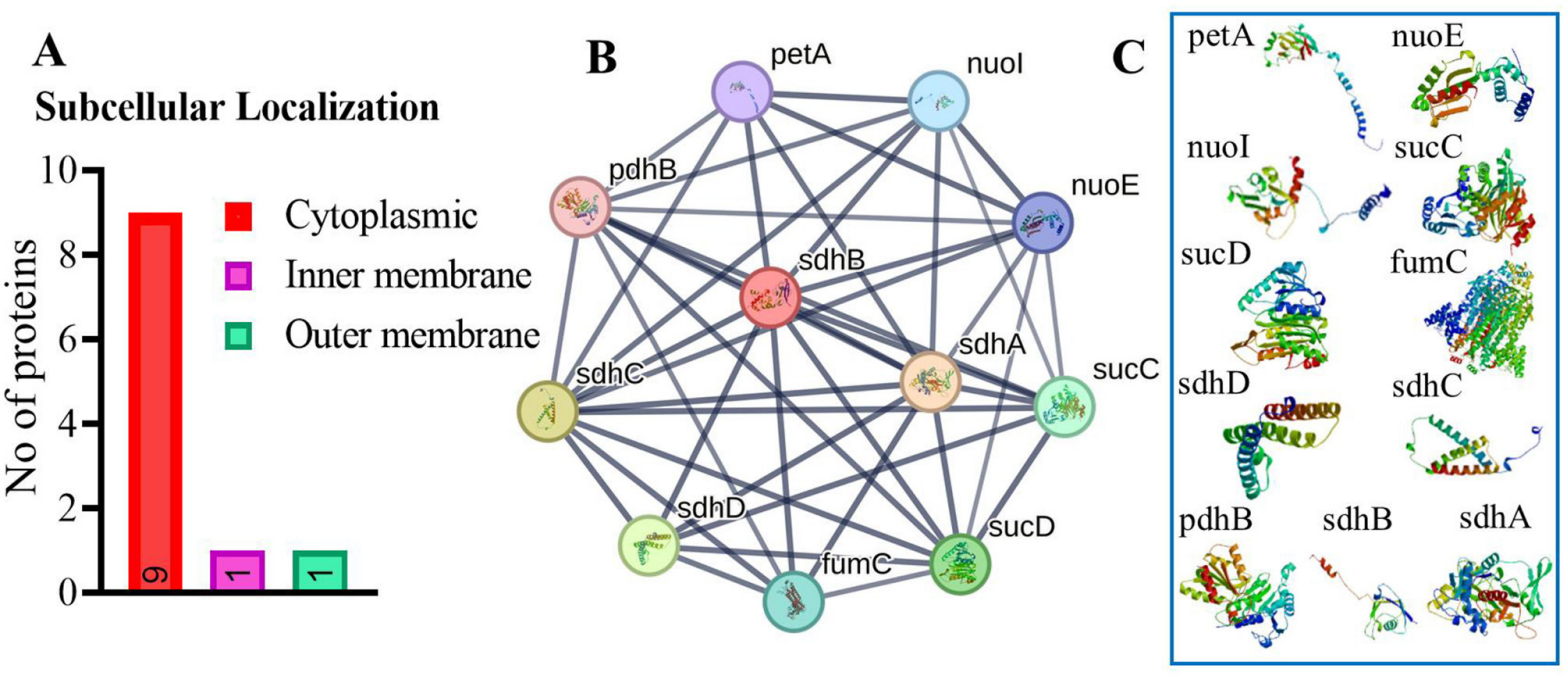
**(A)** Subcellular localization of vital, druggable, and pathogen-specific proteins. **(B)** Protein-protein interaction network for succinate dehydrogenase, showing various protein interactions represented as nodes and edges. Colored nodes indicate the query protein. **(C)** Illustrate the proteins which interact with succinate dehydrogenase.

Our analysis found that 90% of proteins were cytoplasmic, with 5% in both the inner and outer membranes (Figure 2A). The identified target protein, succinate dehydrogenase, was cytoplasmic, indicating its potential as a hub protein due to its extensive interactions. Targeting succinate dehydrogenase could disrupt the function of other interacting proteins, underscoring its critical role in cellular processes (82). The protein-protein interaction (PPI) analysis showed the succinate dehydrogenase interaction with ubiquinol-cytochrome reductase (petA), NADH dehydrogenase I, chain I (nuoI), NADH dehydrogenase I, chain E (nuoE), succinyl-CoA synthase beta chain (SucC), succinyl CoA synthase alpha chain (SucD), fumarate hydratase (fumC), succinate dehydrogenase hydrophobic membrane protein (sdhD), pyruvate dehydrogenase E1 component (pdhB), succinate dehydrogenase cytochrome subunit (sdhC), and succinate dehydrogenase flavoprotein (sdhA). The PPI analysis of succinate dehydrogenase revealed a network with 11 nodes, an average node degree of 8, an average local clustering coefficient of 0.863, and a total of 44 edges (Figure 2B). The PPI enrichment *p*-value was calculated to be 6.51e^−11^, indicating a significant interaction network, with an expected number of 14 edges. These proteins participate in various essential functions, suggesting that inhibiting succinate dehydrogenase could potentially disrupt the activity of other interacting proteins (Figure 2C) (83). This makes succinate dehydrogenase a promising candidate for a drug target.

#### 3.1.5 Succinate dehydrogenase as a new drug target

**Figure 3:**
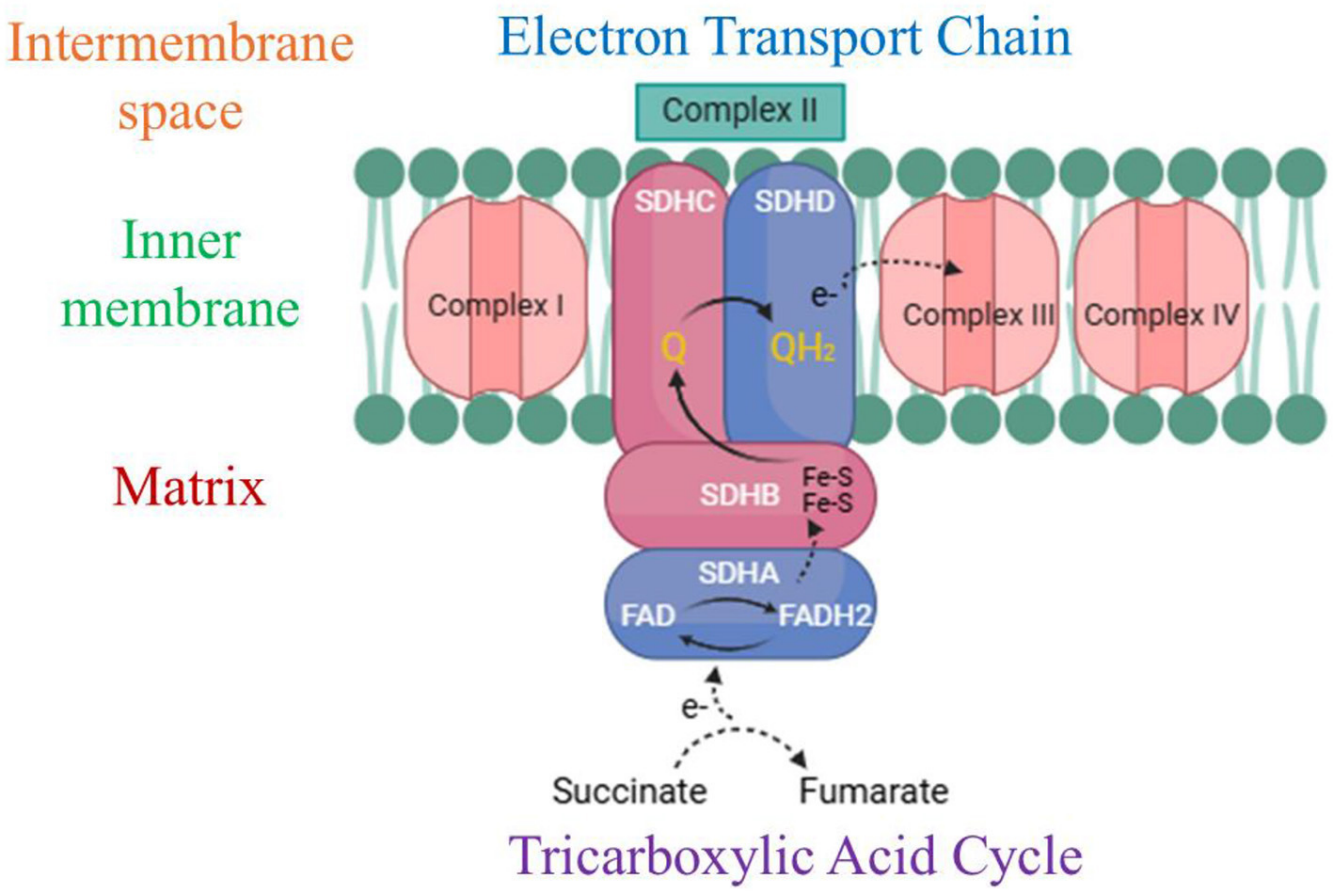
Diagrammatic representation of the succinate dehydrogenase/complex II structure and function. This complex contributes to both the tricarboxylic acid (TCA) cycle and electron transport chain (ETC) by facilitating the electron transfer process from succinate to ubiquinone (Q) via its four integral subunits: SDHA, SDHB, SDHC, and SDHD. Key elements include Fe-S clusters (iron–sulfur clusters), ubiquinone (Q), ubiquinol (QH2), the inner mitochondrial membrane, and the intermembrane space. (Created with https://smart.servier.com/ and Draw.io https://app.diagrams.net/).

The succinate dehydrogenase protein in *R. felis* presents a compelling target for drug development due to its vital role in the tricarboxylic acid (TCA) cycle and electron transport chain (ETC), both essential for ATP production (84). Succinate dehydrogenase (SDH) is a mitochondrial enzyme that plays a pivotal role in cellular metabolism by linking the tricarboxylic acid (TCA) cycle and the electron transport chain (ETC) (85). Within the TCA cycle, succinate dehydrogenase facilitates the oxidation of succinate into fumarate, while in the ETC, it reduces ubiquinone to ubiquinol, functioning as complex II. This enzyme facilitates electron transfer through its four subunits (SDHA, SDHB, SDHC, and SDHD), enabling the movement of electrons from FADH_2_ to ubiquinone and subsequently to complex III, contributing to the production of adenosine triphosphate (ATP), the primary energy currency of the cell. The catalytic subunit SDHA, the largest component of the succinate dehydrogenase (SDH) complex, is responsible for oxidizing succinate into fumarate, producing FADH_2_ in the process as part of the tricarboxylic acid (TCA) cycle (86). The SDHB subunit houses three iron–sulfur clusters that facilitate electron transfer from FADH_2_ to the membrane-embedded subunits SDHC and SDHD. These latter subunits, situated in the inner mitochondrial membrane, form the electron transport chain's (ETC) complex II and serve as the binding and reduction site for ubiquinone (Q) to ubiquinol (QH_2_) (87) (Figure 3). Therefore, its disruption can lead to impaired ATP synthesis, metabolic imbalances, and increased oxidative stress, ultimately weakening the bacterium's ability to thrive and cause infection. Given its critical function in both metabolism and virulence, succinate dehydrogenase emerges as a promising target for therapeutic interventions aimed at inhibiting the survival and proliferation of *R. felis* (88).

#### 3.1.6 3D structure prediction and its validation

**Figure 4:**
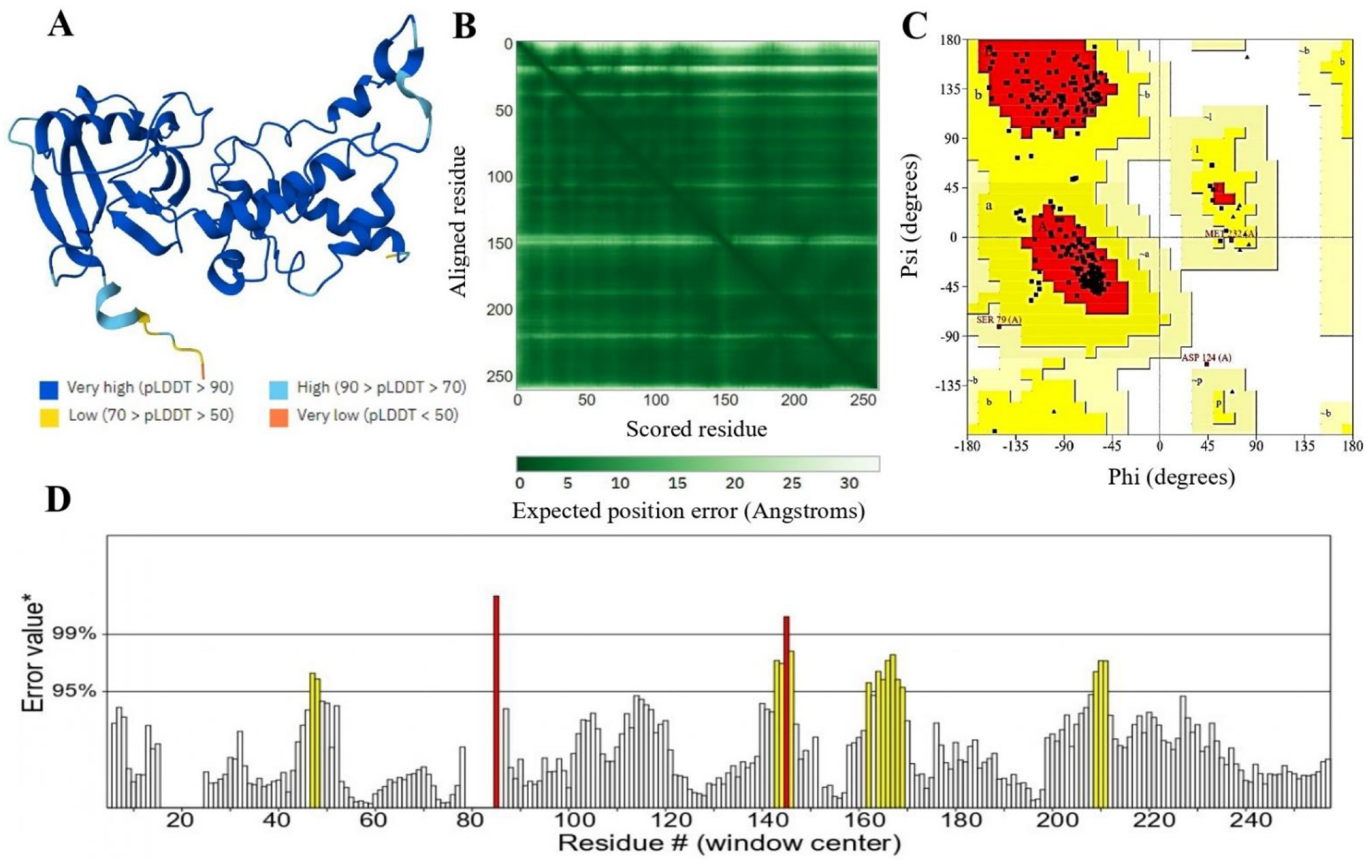
**(A)** The 3D structure of succinate dehydrogenase was modeled using AlphaFold2. AlphaFold2 assigns a confidence score (pLDDT) from 0 to 100 for each residue. Scores below 50 suggest unstructured regions, while higher scores indicate more reliable predictions. **(B)** The anticipated aligned error of the model is depicted, where dark green areas represent low error, and light green areas signify higher error rates. **(C)** Represents 86.3% validity by Ramachandran plot and **(D)** represents 92.7% ERRAT score of the predicted 3D structure.

The protein 3D structure provided by AlphaFold2, includes some regions of disorder, which are indicated by low pLDDT values (89). In the resulting model, most residues exhibit very high confidence scores (pLDDT > 90), indicating the model's accuracy (Figure 4A). The dark green areas in the predicted aligned error plot signify high accuracy, while the light green areas suggest higher error rates (Figure 4B) (90). The structure was validated using the PROCHECK and ERRAT servers which revealed good quality of three-dimensional models (91). Ramachandran plot categorized residues into three regions: favored (86.3%), allowed (12.3%), and disallowed (0.4%) (Figure 4C). Additionally, the ERRAT server evaluated the model's quality based on statistical interactions among non-bonded atoms of different types. ERRAT scores around 92.7% indicate high-resolution structures as shown in Figure 4D.

### 3.2 Molecular docking, virtual screening, and ADMET profiling

#### 3.2.1 Active site identification and ligand prediction for the modeled protein

**Figure 5:**
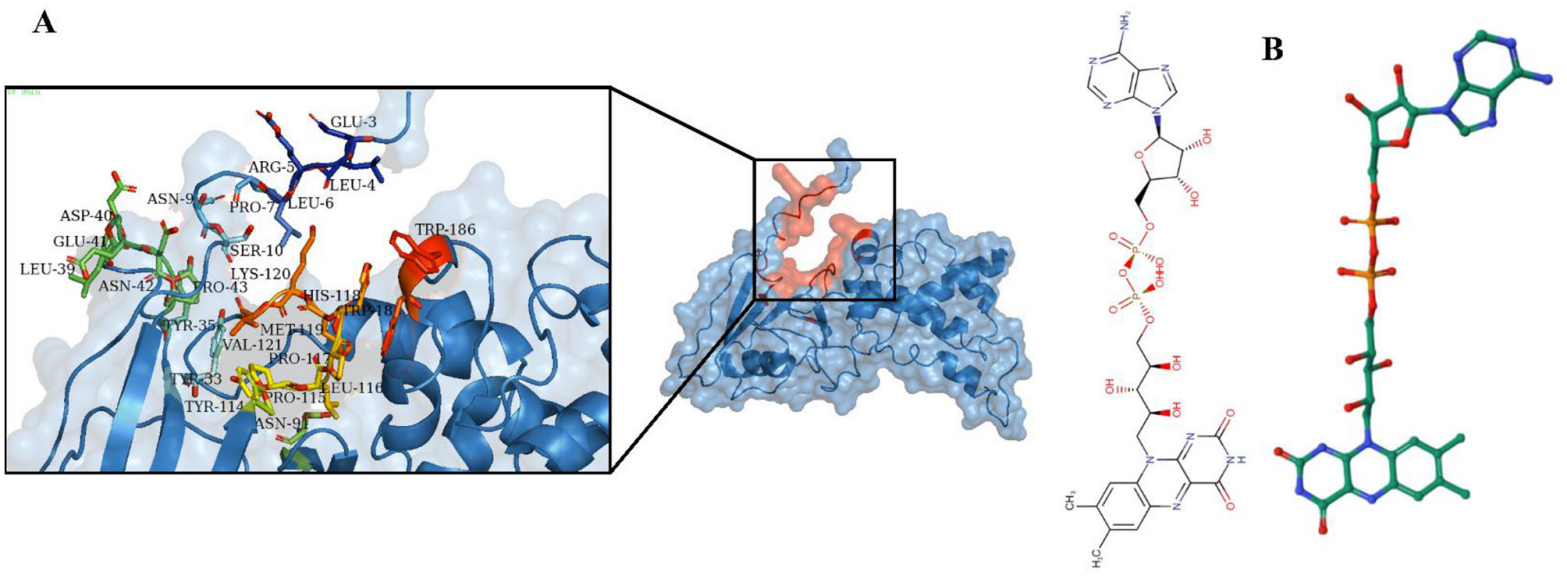
Identification of ligand and active site. **(A)** The active site of the succinate dehydrogenase is determined using MOE. **(B)** The ProBis tool predicts Flavin-adenine dinucleotide (FAD) as a ligand for succinate dehydrogenase.

The functional activity of a protein depends on the binding of a ligand to its active site (92). Identifying this binding site is crucial for understanding the protein's role and for drug discovery (93). MOE utilized the 3D template structure, which shares similarities with known ligand-binding sites, to identify potential binding pockets through geometric analysis (94). Multiple active sites were found, with the site having the highest energy profile and key functional residues selected (Figure 5A). Predicting protein ligands is challenging, as similar protein folds do not always imply similar functions (95). Conversely, proteins with different folds can exhibit similar biochemical properties, emphasizing that the binding site is crucial for protein function (96). The ProBiS revealed high sequence and structural homology with rhodoquinol-fumarate reductase complexed with Flavin-adenine dinucleotide (FAD) (PDB ID 3VR8) (Figure 5B). A lower binding energy, such as that observed here, suggests a stronger binding affinity, which often correlates with effective inhibition of enzyme activity.

#### 3.2.2 Molecular docking and ligand-protein interactions analysis

**Figure 6:**
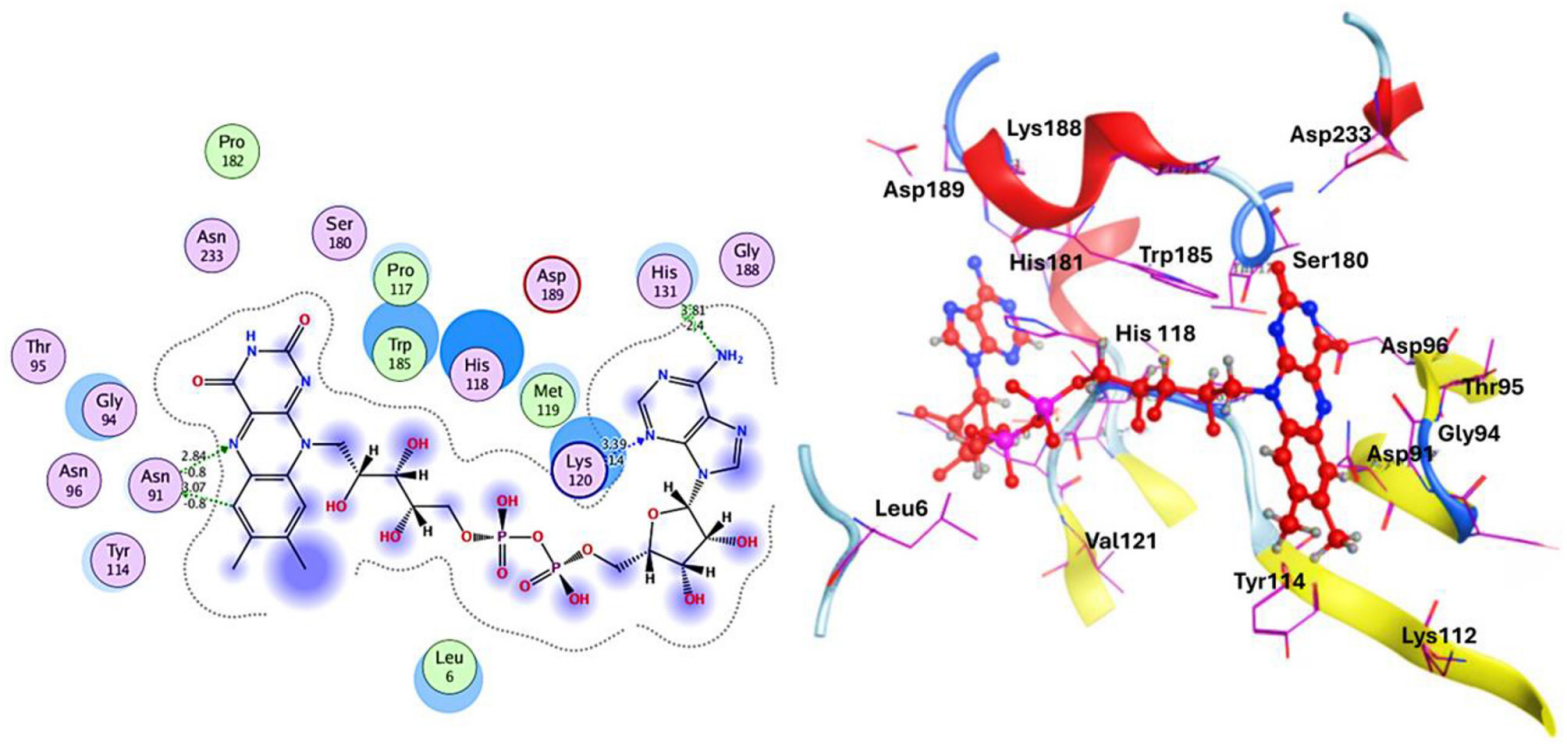
Docking analysis of the succinate dehydrogenase revealed a binding affinity of −8.47 kcal/mol for FAD at the active site.

Various bioinformatics tools are used for molecular docking in drug discovery (97). MOE module provides detailed graphical representations and ranks receptor-ligand binding affinities using the S-score, a measure of binding free energy (in kcal/mol), where a lower score indicates a more favorable binding (98). Docking of Flavin-adenine dinucleotide (FAD) to succinate dehydrogenase revealed five distinct conformations. Conformation 1, with a high binding energy of −8.47 kcal/mol, was selected for further analysis due to its superior binding affinity compared to the lowest score of −7.50 kcal/mol. This indicates that conformation 1 represents the most stable and potentially effective binding mode for FAD, supporting its role in the protein's function (Figure 6, Table 2) (99).

**Table 2:** Protein-ligand interaction and docking score.

**Compound ID**	**Docking score**	**Amino acid**			**Interactions**	**Distance**	**E (kcal/mole)**
DB03147	−8.47	OD1	ASN	91	H-donor	3.07	−0.8
		N	LYS	120	H-acceptor	3.39	−1.4
		ND2	ASN	91	H-acceptor	2.84	−0.8
		5-ring	HIS	131	pi-H	3.81	−2.4

**Figure 7:**
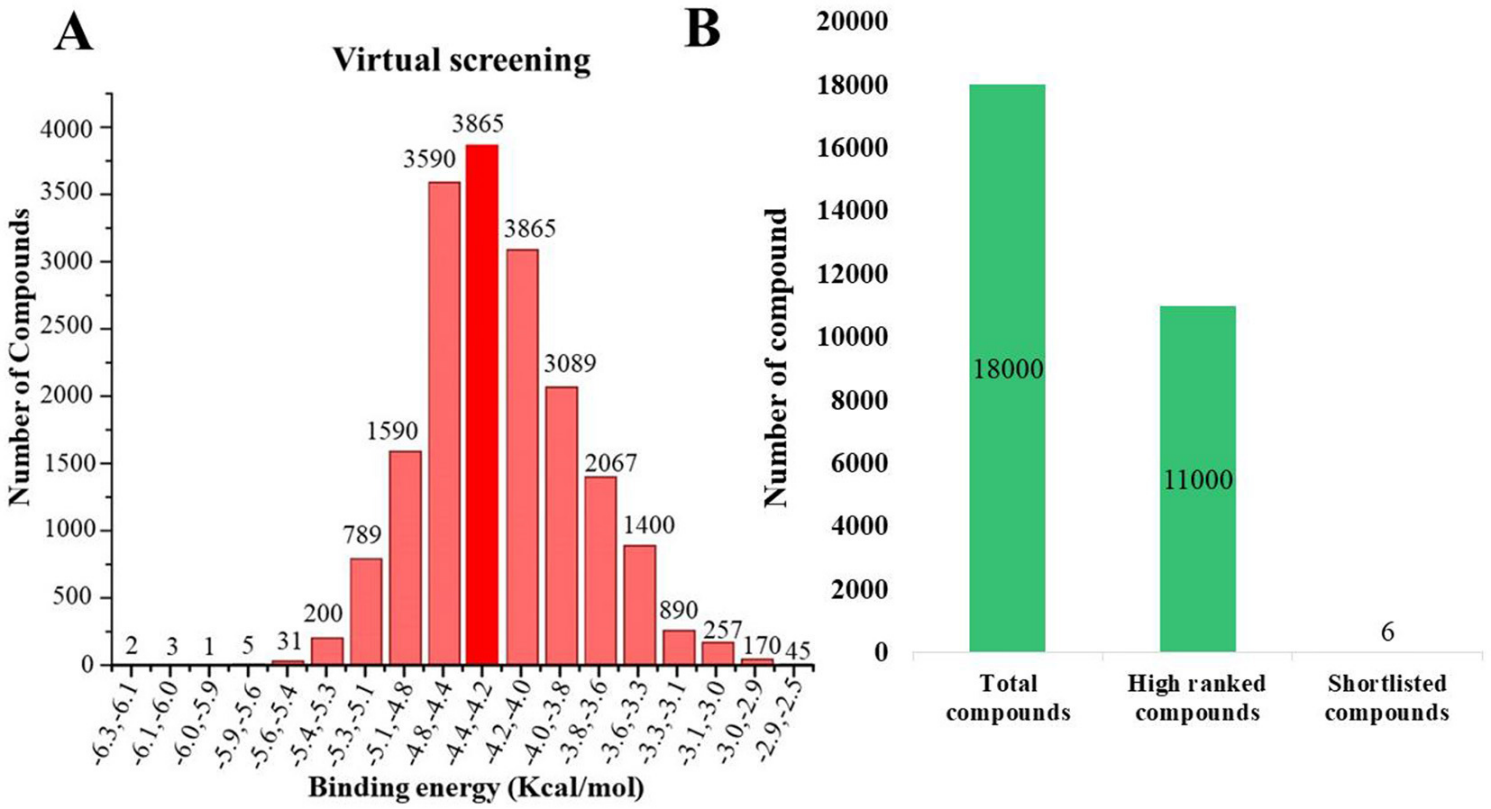
**(A)** Virtual screening was conducted for succinate dehydrogenase using a ZINC database containing 18,000 compounds, revealing binding affinities ranging from −2 to −6 kcal/mol. The analysis identified approximately 3,865 compounds that docked at the same binding site with energies between −4.4 and −4.2 kcal/mol (depicted in dark red). **(B)** An overview of the virtual screening process is presented, narrowing down from an initial pool of 18,000 compounds to 11,000 top-ranked candidates. This ultimately led to the discovery of six potential inhibitors: ZINC67847806, ZINC67982856, ZINC67974679, ZINC67895371, ZINC05668040, and ZINC05670149.

A comprehensive *in silico* screening of the 18,000-compound ZINC library was conducted against the active site of succinate dehydrogenase using an intensive docking approach. This process generated numerous docked conformations, ranked according to their docking scores. Compounds exhibiting lower binding affinities than the reference inhibitor, FAD with a binding energy threshold of −8.47 kcal/mol, were eliminated from further consideration as potential hit candidates. The screening identified over 11,000 compounds with binding energies surpassing that of FAD, ranging between −6.32 kcal/mol and −4.42 kcal/mol (Figure 7A, indicated in dark red). These results suggest that the inhibition of succinate dehydrogenase by these molecules could represent promising leads, as the favorable binding energies imply that these compounds form stable interactions with succinate dehydrogenase, which could effectively inhibit its function (100). Compounds demonstrating higher binding affinities than the FAD inhibitor were prioritized for further investigation due to their potent inhibitory potential against succinate dehydrogenase (Figure 7B). From this group, six compounds emerged as viable therapeutic candidates against succinate dehydrogenase in *R. felis*: ZINC67847806, ZINC67982856, ZINC67974679, ZINC67895371, ZINC05668040, and ZINC05670149 which were used for further analysis.

#### 3.2.3 Post-docking interaction analysis of selected compounds with succinate dehydrogenase

**Figure 8:**
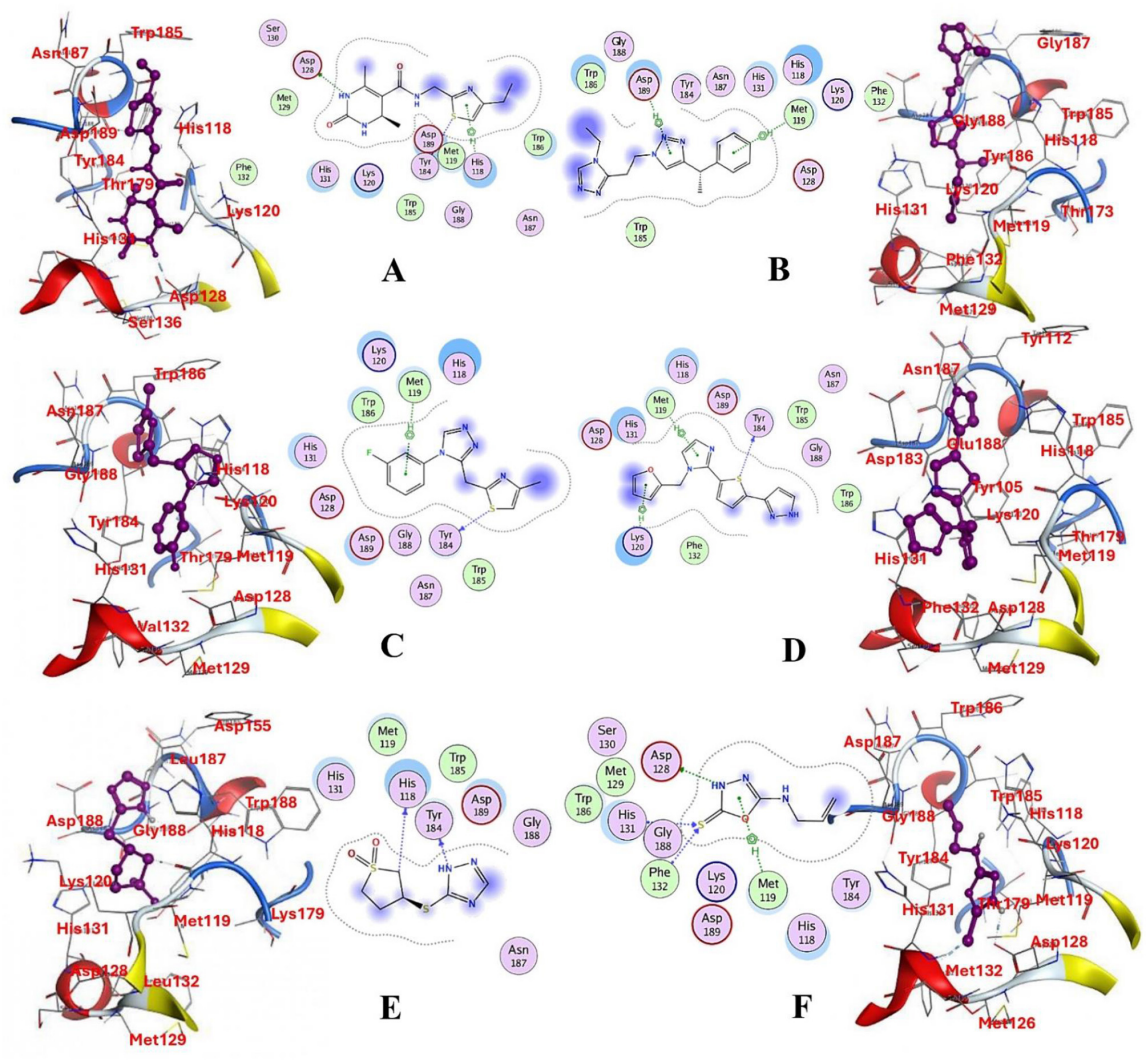
Molecular docking analysis for the shortlisted compound **(A)** ZINC67974679, **(B)** ZINC67895371, **(C)** ZINC67982856, **(D)** ZINC67847806, **(E)** ZINC05670149, **(F)** ZINC05668040.

The selected compounds were subjected to post-docking interaction analysis to gain further insights into the pharmacological characteristics and binding dynamics of succinate dehydrogenase. Multiple interactions between each ligand and the receptor were observed during the molecular docking analysis. The docking rank order, based on binding scores, is as follows: ZINC67974679 > ZINC67895371 > ZINC67982856 > ZINC67847806 > ZINC05670149 > ZINC05668040. In the docking study, ZINC67974679 demonstrated a binding energy of −6.54 kcal/mol. The compound's five-aromatic ring facilitated the formation of one pi interaction with HIS118 achieved binding energy of −0.8 kcal/mol, and two H-donor interactions with TYR184 and ASP128, with bond lengths of 3.71 and 3.07 Å, and interaction energy (E) of −0.9 and −6.3 kcal/mol, respectively (Figure 8A). ZINC67895371 achieved a docking score of −6.40 kcal/mol. The six and five aromatic rings of this compound established a pi-hydrogen bond with MET119 and ASP189, characterized by a bond length of 3.78 Å, 3.95 and an energy of −0.6 and −0.7 kcal/mol (Figure 8B). ZINC67982856 was highly compatible with the succinate dehydrogenase binding site, displaying a binding score of −6.02 kcal/mol. The six aromatic rings engaged in one H-donor and a pi-H interaction with TYR184 and MET119, with bond lengths ranging from 3.64 to 4.43 Å and interaction energies between −0.7 and −0.9 kcal/mol (Figure 8C). ZINC67847806 formed one hydrogen bond as a donor with TYR184, resulting in a binding energy of −6.00 kcal/mol with bond lengths ranging from 3.97 to 3.75 Å and interaction energies between −0.7 and −0.8 kcal/mol (Figure 8D). Conversely, ZINC05670149 initiated two hydrogen bonds as donors from TYR184 and HIS118. This compound demonstrated a binding score of −5.27 kcal/mol (Figure 8E). Finally, ZINC05668040 docked with succinate dehydrogenase, producing a binding energy of −4.73 kcal/mol. The compound's aromatic ring mediated a single pi-hydrogen bond with the MET119, and three hydrogen bonds as acceptors (HIS131, PHE132), and one hydrogen bond as a donor with ASP128 residue (Figure 8F). Compounds with lower docking scores, such as ZINC67974679 and ZINC67895371, are expected to show increased stability within the binding site and thus greater inhibitory potential, supporting their prioritization for further investigation. The lower binding energies observed in these interactions suggest that these compounds form stable complexes with succinate dehydrogenase, potentially disrupting the enzyme's activity effectively (101). This correlation between docking score and inhibitory potential underscores these candidates' potential efficacy. The detailed binding interactions within succinate dehydrogenase active site for the selected compounds is presented in Table 3.

**Table 3:** Docking scores and identified bond types for the selected compounds were determined through the MOE tool.

**Compound ID**	**Docking score**	**Receptors**	**Interactions**	**Distance**	**E (kcal/mole)**
ZINC67974679 5-ring	−6.54	O TYR 184	H–donor	3.71	−0.9
		OD2 ASP 128	H–donor	3.07	−6.3
		NE2 HIS 118	pi–H	3.74	−0.8
ZINC67895371 6-ring 5-ring	−6.40	CG MET 119	pi–H	3.78	−0.6
		CB ASP 189	pi–H	3.95	−0.7
ZINC67982856 6-ring	−6.02	O TYR 184	H–donor	3.64	−0.7
		CA MET 119	pi–H	4.43	−0.9
ZINC67847806 S1 16 5-ring 5-ring	−6.00	O TYR 184	H–donor	4.33	−0.5
		CG MET 119	pi–H	3.97	−0.7
		CD LYS 120	pi–H	3.75	−0.8
ZINC05670149 N2 4 C6 15	−5.27	O TYR 184	H–donor	3.45	−0.8
		O HIS 118	H–donor	3.24	−1.2
ZINC05668040 N3 10 S1 10 S1 10 5-ring	−4.73	OD2 ASP 128	H–donor	3.33	−1.6
		N HIS 131	H–acceptor	3.41	−2.0
		N PHE 132	H–acceptor	3.28	−2.8
		CG MET 119	pi–H	3.71	−1.6

#### 3.2.4 Pharmacokinetic assessment

A pivotal aspect of drug discovery is the examination of ADMET properties, as it significantly reduces time and expenses during clinical trials (102). The pharmacokinetic evaluation revealed key insights into the drug-likeness, ADME characteristics, and blood-brain barrier (BBB) penetration of the selected compounds (103). The Lipinski Rule of Five (RO5) served as the foundation for determining drug-likeness, stipulating that drug-like molecules should possess a molecular weight under 500 Daltons, with a maximum of 10 hydrogen bond acceptors and <5 hydrogen bond donors and a logP (lipophilicity) value below 5 (104). Following these criteria, compounds ZINC20115475, ZINC02688148, and ZINC04259566 satisfied the RO5, whereas ZINC95543764, ZINC04232055, and ZINC04231816 exhibited a single violation each. Nonetheless, all six compounds were determined to lack BBB permeability, which evaluates the compound's ability to cross the protective blood-brain barrier to reach the central nervous system. The compounds adhering to drug-likeness criteria progressed to the subsequent phase of the study. For preliminary ADME property estimation, the pkCSM tool was utilized (105). This analysis encompassed solubility in pure water (mg/L), gastrointestinal absorption (HIA), which indicates the compound's potential for oral absorption in the intestine, permeability, inhibition of liver enzymes such as CYP 2C19, CYP 2C9, CYP 2D6, and CYP 3A4, as well as Caco-2 cell permeability, a model used to predict intestinal drug absorption and permeability through cell monolayers (106). The water solubility of these compounds ranged from −2 to −5, with ZINC02688148 exhibiting high solubility. Caco-2 permeability values spanned from −0.025 to 0.94, with ZINC04259566 demonstrating high cell permeability and ZINC95543764 the lowest. The high permeability observed for ZINC04259566 aligns with studies suggesting that compounds with permeability values exceeding 0.5 tend to exhibit favorable intestinal absorption profiles, making them promising candidates for oral drug delivery (77). Additionally, all compounds displayed good potent HIA permeability, leading to the recommendation of ZINC67982856 for further experimental validation (Table 4). Moreover, an Ames mutagenicity test conducted using the pkCSM tool assessed the potential toxicity of these compounds, including maximum tolerated dose (human), minnow toxicity, *T. pyriformis* toxicity, oral rat acute toxicity (LD50), hepatotoxicity, and skin sensitization (107). The use of *T. pyriformis* as a model organism in toxicity studies is due to its sensitivity to chemical compounds, providing valuable insights into aquatic toxicity (106). All compounds except ZINC20115475 yielded a negative Ames test, indicating a lack of mutagenic potential. The LD50 values ranged from 2.8 to 4.3 mol/kg, with ZINC02688148 exhibiting the highest value, indicating lower acute oral toxicity in rats. These results are consistent with other studies where compounds with higher LD50 values demonstrated greater safety margins, particularly for therapeutic agents intended for prolonged use (108). The data for oral rat acute toxicity (LD50), hepatotoxicity, and skin sensitization were predicted computationally using the pkCSM tool, which evaluates toxicity based on chemical structure and quantitative structure-activity relationships (QSAR). Given that a chemical's toxicity is often predicted based on its molecular structure, ZINC20115475 was excluded from further studies due to its positive result in the Ames test, suggesting possible mutagenicity. While ZINC02688148, ZINC04259566, ZINC04232055, and ZINC04231816 were found to be hepatotoxic, none of the compounds induced skin sensitization. The hepatotoxic compounds were not considered for future investigation, as previous study indicate the dose-dependent liver toxicity profiles and mitigate potential risks (109). *T. pyriformis* exhibited maximum tolerance to ZINC20115475 (0.367 log μg/L), whereas the remaining compounds were less tolerated. Detailed information can be found in Table 5.

**Table 4:** ADME analysis of the shortlisted compounds against succinate dehydrogenase.

**ZINC ID**	**Water solubility (log mol/L)**	**CaCo2** ** permeability (log Papp in 10-6 cm/s)**	**Intestinal absorption (human)**	**Skin permeability (log Kp)**	**BBB permeability (log BB)**	**Structure**
						
ZINC67974679	−3.373	0.69	77.32	−3.695	−0.973	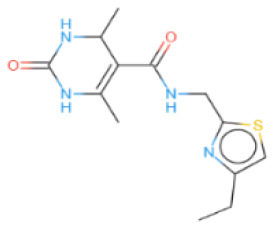
						
ZINC67895371	−3.162	1.247	98.70	−2.661	−0.123	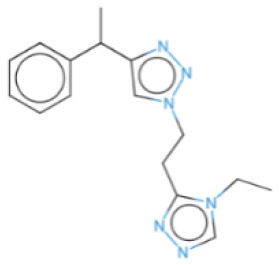
						
ZINC67982856	−3.45	1.327	99.92	−2.515	0.245	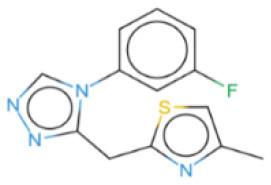
						
ZINC67847806	−2.835	1.341	90.18	−2.735	0.623	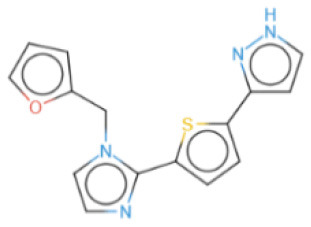
						
ZINC05670149	−1.957	0.571	90.13	−3.124	−1.417	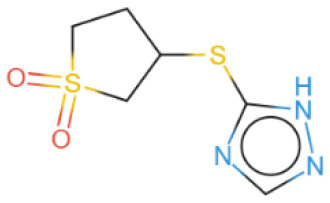
						
ZINC05668040	−2.184	1.155	91.39	−3.182	−0.414	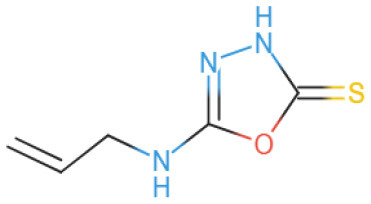

**Table 5:** Toxicity analysis of the shortlisted compounds against succinate dehydrogenase.

**ZINC ID**	**Max. tolerated dose (human)**	**Minnow toxicity (logmM)**	***T. pyriformis* toxicity (log μg/L)**	**Oral rat acute toxicity (LD50) (mol/kg)**	**Ames test**	**Hepatotoxicity**	**Skin sensitization**
ZINC67974679	0.513	2.815	0.061	2.632	No	No	No
ZINC67895371	0.561	−0.691	0.431	2.212	Yes	Yes	No
ZINC67982856	−0.522	0.944	0.514	2.316	No	No	No
ZINC67847806	0.099	1.968	0.285	2.306	Yes	Yes	No
ZINC05670149	−0.334	2.993	0.069	2.492	No	Yes	No
ZINC05668040	0.892	2.768	−0.399	2.794	No	No	No

## 4 Conclusions

This study employed the subtractive proteomics method to prioritize viable drug targets against the *R. felis*. The approach involves several essential analyses at different stages, including identifying non-host homologous, essential, druggable, and pathogen-specific proteins. Several proteins, including succinate dehydrogenase, were identified as novel drug targets against *R. felis*. The selected essential proteins could serve as therapeutic targets for the development of new drugs or vaccines against *R. felis*. Additionally, a pharmacoinformatic approach was utilized to screen a natural product's ZINC library (n = 18,000) against succinate dehydrogenase for potential inhibitors. Six compounds— ZINC67847806, ZINC67982856, ZINC67974679, ZINC67895371, ZINC05668040, and ZINC05670149—were identified as promising inhibitors based on their ligand-protein binding patterns (lowest estimated binding energy). However, ADMET profiling indicated that while all compounds generally met the required ADMET properties, ZINC67895371 and ZINC67847806 showed positive Ames activity, and ZINC05670149, ZINC67895371, and ZINC67847806 exhibited hepatotoxicity, though none showed skin sensitization. Based on these findings, we recommend the ZINC67974679, ZINC67982856, and ZINC05668040 compounds for further experimental validation. Nonetheless, experimental validation is needed to enhance the efficacy of the predicted targets.

## Data Availability

The original contributions presented in the study are included in the article/Supplementary material, further inquiries can be directed to the corresponding authors.
