# Supplementary material for: Prediction of potential drug targets and key inhibitors (ZINC67974679, ZINC67982856, and ZINC05668040) against Rickettsia felis using integrated computational approaches
Source: Front Vet Sci. 2025 Jan 16;11:1507496. doi: 10.3389/fvets.2024.1507496 (PMC11780677; doi:10.3389/fvets.2024.1507496)
Supplement: Supplementary file 1 [file Table_1.docx]

Supplementary Material

**Supplementary table S1:** List of unique metabolic pathways in *R. felis*.

| **S.no** | **Pathway ID** | **Pathway Name** |
| --- | --- | --- |
| 1 | rfe00074 | Mycolic acid biosynthesis |
| 2 | rfe00261 | Monobactam biosynthesis |
| 3 | rfe00300 | Lysine biosynthesis |
| 4 | rfe00362 | Benzoate degradation |
| 5 | rfe00460 | Cyanoamino acid metabolism |
| 6 | rfe00521 | Streptomycin biosynthesis |
| 7 | rfe00540 | Lipopolysaccharide biosynthesis |
| 8 | rfe00541 | O-antigen nucleotide sugar biosynthesis |
| 9 | rfe00550 | Peptidoglycan biosynthesis |
| 10 | rfe00552 | Teichoic acid biosynthesis |
| 11 | rfe00680 | Methane metabolism |
| 12 | rfe00710 | Carbon fixation by Calvin cycle |
| 13 | rfe00720 | Other carbon fixation pathways |
| 14 | rfe00907 | Pinene, camphor, and geraniol degradation |
| 15 | rfe00930 | Caprolactam degradation |
| 16 | rfe00946 | Degradation of flavonoids |
| 17 | rfe01110 | Biosynthesis of secondary metabolites |
| 18 | rfe01120 | Microbial metabolism in diverse environments |
| 19 | rfe01501 | beta-Lactam resistance |
| 20 | rfe01502 | Vancomycin resistance |
| 21 | rfe01503 | Cationic antimicrobial peptide (CAMP) resistance |
| 22 | rfe02020 | Two-component system |
| 23 | rfe02024 | Quorum sensing |
| 24 | rfe03060 | Protein export |
| 25 | rfe03070 | Bacterial secretion system |

**Supplementary table S2:** Unique metabolic proteins are involved in unique metabolic pathways.

| **S.no** | **Pathway Name** | **KO Codes** | **Pathway Name** |
| --- | --- | --- | --- |
|  | WP_011270515.1 | K00626 | Benzoate degradation |
|  | WP_011271493.1 | K00600 | Cyanoamino acid metabolism |
|  | WP_011270920.1 | K00024 | Methane metabolism |
|  | WP_011270490.1 | K00240 | Carbon fixation pathways |
|  | WP_011270625.1 | K01679 | Carbon fixation pathways |
|  | WP_011270995.1 | K01903 | Carbon fixation pathways |
|  | WP_011271564.1 | K01681 | Carbon fixation pathways |
|  | WP_039594975.1 | K01902 | Carbon fixation pathways |
|  | WP_039595039.1 | K01491 | Carbon fixation pathways |
|  | WP_039595177.1 | K00239 | Carbon fixation pathways |
|  | WP_011271325.1 | K00626 | Two-component system |
